# Human Polyomaviruses in the Cerebrospinal Fluid of Neurological Patients

**DOI:** 10.3390/microorganisms8010016

**Published:** 2019-12-20

**Authors:** Serena Delbue, Diego Franciotta, Sara Giannella, Maria Dolci, Lucia Signorini, Rosalia Ticozzi, Sarah D’Alessandro, Giuseppina Campisciano, Manola Comar, Pasquale Ferrante, Marco Ciotti

**Affiliations:** 1 Department of Biomedical, Surgical and Dental Sciences, University of Milano, Via Pascal, 36, 20133 Milano, Italy; serena.delbue@unimi.it (S.D.); maria.dolci@unimi.it (M.D.); lucia.signorini@unimi.it (L.S.); rosalia.ticozzi@unimi.it (R.T.); sarah.dalessandro@unimi.it (S.D.); 2 Neuroimmunology Laboratory, IRCCS Mondino Foundation, Via Mondino 2, 27100 Pavia, Italy; diego.franciotta@mondino.it; 3 Virology Unit, Laboratory of Microbiology and Virology, Polyclinic Tor Vergata Foundation, Viale Oxford 81, 00133 Rome, Italy; saraxgiannella@hotmail.com (S.G.); marco.ciotti@ptvonline.it (M.C.); 4 Institute for Maternal and Child Health—IRCCS “Burlo Garofolo”, 34137 Trieste, Italy; g.campisciano@gmail.com (G.C.); manola.comar@burlo.trieste.it (M.C.); 5 Department of Medical Sciences, University of Trieste, 34149 Trieste, Italy

**Keywords:** human polyomaviruses, cerebrospinal fluid, neurological diseases

## Abstract

Background: Central nervous system (CNS) infections by human polyomaviruses (HPyVs), with the exception of JC (JCPyV), have been poorly studied. Methods: In total, 234 cerebrospinal fluid (CSF) samples were collected from patients affected with neurological disorders. DNA was isolated and subjected to quantitative real-time PCR (Q-PCR) for the detection of six HPyVs: JCPyV, BKPyV, Merkel cell PyV (MCPyV), HPyV6, HPyV7, and HPyV9. Where possible, the molecular characterization of the viral strains was carried out by nested PCR and automated sequencing. Results: JCPyV was detected in 3/234 (1.3%), BKPyV in 15/234 (6.4%), MCPyV in 22/234 (9.4%), and HPyV6 in 1/234 (0.4%) CSF samples. JCPyV was detected at the highest (*p* < 0.05) mean load (3.7 × 10^7^ copies/mL), followed by BKPyV (1.9 × 10^6^ copies/mL), MCPyV (1.9 × 10^5^ copies/mL), and HPyV6 (3.3 × 10^4^ copies/mL). The noncoding control regions (NCCRs) of the sequenced viral strains were rearranged. Conclusions: HPyVs other than JCPyV were found in the CSF of patients affected with different neurological diseases, probably as bystanders, rather than etiological agents of the disease. However, the fact that they can be latent in the CNS should be considered, especially in immunosuppressed patients.

## 1. Introduction

The *Polyomaviridae* family comprises small, naked, DNA viruses, sharing the same structure. In particular, they have a circular DNA genome of approximately 5 kb divided into a region encoding the early, functional proteins, small and large tumor antigens, a region encoding the late, structural proteins, VP1 and VP2, and a noncoding control region (NCCR), containing the origin of replication and the promoters. The VP1 region is prone to point mutations and is useful for viral genotyping, while the NCCR is a hypervariable region that is susceptible to mutation and heterogeneous rearrangement, leading to pathogenic consequences [1].

Polyomaviruses infect a broad spectrum of hosts, including humans. Since 1971, at least 14 human polyomaviruses (HPyV) have been identified in different biological specimens (cerebrospinal fluid, respiratory samples, skin, serum, stool, and muscle biopsies) [2,3,4,5,6,7,8,9,10,11,12,13,14,15]. Primary HPyVs infection usually occurs in childhood, and is followed by the establishment of an asymptomatic latency state, possibly in the lymphoid, neuronal, kidney or hematopoietic tissues, characterized by low-level replication and shedding, for example, in urine [16]. Seroprevalence studies have shown that the majority of HPyVs, are widespread among world populations, with a seroprevalence of 60%–100% [17]. In healthy individuals, infection is asymptomatic; however, HPyVs can reactivate and cause pathologies in immunocompromised patients [17].

So far, a link with human diseases has been established for six of the identified HPyVs. JC polyomavirus (JCPyV) is the etiological agent of the demyelinating disease of the central nervous system (CNS), progressive multifocal leukoencephalopathy (PML) [18]; BK polyomavirus (BKPyV) has been linked to nephropathy (PVAN) in kidney transplant patients and to hemorrhagic cystitis in hematopoietic stem cell transplant patients [19,20]; Merkel cell polyomavirus (MCPyV) to Merkel cell carcinoma (MCC); Trichodysplasia spinulosa polyomavirus (TSPyV) to trichodysplasia spinulosa, especially in children after kidney transplantation [6]; and human polyomaviruses 6 and 7 (HPyV6 and HPyV7) to pruritic rash [21,22].

As stated, the CNS tropism of JCPyV is very well defined, and BKPyV is occasionally found in both brain tissue and the cerebrospinal fluid (CSF) [23,24]. In contrast, CNS infections by other HPyVs, have been poorly studied, and data regarding the presence of other HPyVs in the CSFs are almost absent from the literature [25,26,27,28,29,30]. Among the published papers, only Dang et al. [27] and Delbue et al. [29] have described the sporadic finding of MCPyV and HPyV6 in the CSF.

Thus, we decided to analyze the presence of MCPyV and HPyV6, 7, and 9, in addition to JCPyV and BKPyV, genomes in CSF samples collected from a large cohort of patients affected by different neurological diseases.

The final aim was to assess the possible relevance of other HPyVs, besides JCPyV and BKPyV, in neurological disorders mostly not related to other microbial agents.

## 2. Materials and Methods

### 2.1. Study Group

In this retrospective, observational study, CSF leftovers after routine laboratory tests were collected and stored at −70 °C from 234 patients, 175 of whom were admitted at the University Hospital Tor Vergata, Rome, and 59 admitted at the Neurological Institute Mondino, Pavia, Italy, from March 2016 to November 2017. Among this group, we included patients with meningoencephalitis (139, 59.4%) and patients with other neurological conditions: peripheral neuropathies (30, 13%), cognitive disorders (19, 8.1%), multiple sclerosis (10, 4.2%), brain tumor (9, 3.8%), epilepsy (5, 2.1%), confused state (5, 2.1%) hydrocephalus (4, 1.7%), migraine (4, 1.7%), myelitis (3, 1.3%), and mood disorders (3, 1.3%). CSF collection was performed at the time of the first diagnosis. Paired sera were also collected for the 59 cases admitted at the Neurological Institute Mondino. Other demographic data are reported in Table 1.

All CSF samples were subjected to testing for the presence of human herpesviruses (herpes simplex virus-1 and -2 (HSV-1 and -2)), varicella zoster virus (VZV), Epstein–Barr virus (EBV), human cytomegalovirus (HCMV), and enteroviruses for diagnostic purposes. Four CSF samples were also subjected to HIV testing, because of signs of cognitive deterioration in HIV+ patients.

The study was approved by the local Ethics Committee Fondazione PTV Policlinico Tor Vergata Protocol no. 211/17, date 08 January 2018 and all patients signed a free and informed consent form during clinician examination.

### 2.2. DNA Isolation

DNA isolation was performed from 150 μL of CSF and from the available sera using the spin-column technique (Nucleospin virus, Macherey-Nagel, Duren, Germany) according to the manufacturer’s instructions. All laboratory procedures were carried out under stringent conditions to avoid contamination, as follows: samples were processed individually; DNA isolation was performed one sample at a time in a clean, dust-free area with sterile and disposable materials, with a change of gloves for each sample.

### 2.3. HPyV Quantitative Real-Time PCR (Q-PCR)

HPyV Q-PCR assays were conducted with DNA isolated from the CSF. The Q-PCR protocols for the detection of JCPyV, BKPyV, MCPyV, HPyV6, HPyV7, and HPyV9 DNA have been previously published [31,32,33,34], and primer and probe sequences are provided in the Supplementary Materials Table S1. PCRs were performed in triplicate in a 20 μL (final volume) reaction mix containing 5 μL of nucleic acid or sterile water in the negative controls. Standard curves were constructed using serially diluted plasmids (10^5^–10 copies/μL) containing whole genomes of JCPyV, BKPyV, HPyV6, and HPyV7 (Addgene, Watertown, MA, USA) and partial genomes of MCPyV, kindly provided by H. Feng, and HPyV9, kindly provided by M.C.W. Feltkamp. The limit of detection was 2 copies/reaction for JCPyV and BKPyV and 10 copies/reaction for MCPyV, HPyV6, HPyV7, and HPyV9.

Only the serum samples paired with positive CSF were tested for the presence of HPyVs. Thus, Q-PCR for the detection of MCPyV and/or BKPyV was conducted on 17 serum samples paired with 17 MCPyV- or BKPyV-positive CSF samples.

Although the CSF was spun before DNA isolation, we hypothesized that some cells may remain. To determine the percentage of infected cells, a concomitant Q-PCR assay targeting the β-globin gene was performed on the HPyV-positive samples. The β-globin gene was amplified using a previously published primer set and the thermal cycling conditions [35]. The results were analyzed by the absolute quantification method, and the data were expressed as HPyV copies/mL of CSF and as a percentage of infected cells calculated as follows: ([viral copies/mL]/[β-globin copies/2/mL]) × 100.

### 2.4. JCPyV, BKPyV and MCPyV Molecular Characterization

Molecular characterization of the positive strains was possible in only some cases because of a lack of specimens. CSF samples with positive results for the presence of BKPyV and/or JCPyV genomes were molecularly characterized using nested PCR for the VP1 genotyping fragment and NCCR, as previously described [36]. Additionally, the CSF samples with positive results for the presence of the MCPyV genome were further characterized using nested PCR for the NCCR region, in accordance with the protocol published by Hashida and colleagues [37]. The DNA sequencing of both strands of the PCR fragments was carried out at an external facility (Eurofins Genomics, Germany). BLAST searches on the NCBI site (https://blast.ncbi.nlm.nih.gov) were used to determine the sequence homology, as previously described by Agostini et al. [38] for JCPyV genotyping, and by Ault et al. [39] and Jensen and Major [40] for JCPyV NCCR rearrangements. The BKPyV genotype was determined in accordance with the classification method proposed by Jin et al. [41]. The MCPyV NCCRs were aligned with those of the prototype strain MCC350 deposited in GenBank under the accession number EU375803.

### 2.5. Statistical Analysis

The χ^2^ test and the Fisher exact test were used to evaluate the significance of the association among HPyV infection, HPyV load, and HPyV infection and neurological disease; *p* < 0.05 was considered significant.

## 3. Results

### 3.1. HPyV Detection by Q-PCR

Overall, HPyV genomes were detected in 41/234 (17.5%) CSF samples. Of the six searched HPyVs, JCPyV was detected in 3/234 (1.3%), BKPyV in 15/234 (6.4%), MCPyV in 22/234 (9.4%), and HPyV6 in 1/234 (0.4%) CSF samples. BKPyV and MCPyV were significantly more frequently detected than JCPyV (*p* < 0.05). Additionally, three CSF samples were positive for the presence of both BKPyV and MCPyV genomes, as reported in Table 2. None of the CSF samples were positive for HPyV7 or HPyV9. Some HPyV-positive CSF samples were also positive for the other viruses, which were tested for diagnostic purposes. Notably, EBV was detected in two JCPyV-positive CSF samples and in two BKPyV-positive CSF samples; HSV-1 was found in one BKPyV- and one MCPyV-positive CSF sample; HIV was amplified in two JCPyV-positive CSF samples, and enteroviruses were found in one BKPyV-positive and in three MCPyV-positive CSF samples, as seen in Table 3. Detailed viral loads for viruses other than HPyVs are described in the Supplementary Materials.

Where possible, sera of patients with CSF positive for HPyVs were tested. One sample out of 17 tested was positive for the presence of the BKPyV genome, with a viral load of 6.05 × 10^2^ copies/mL, corresponding to a CT of 39.02.

### 3.2. HPyV Load and Infected Cells Percentage

**Figure 1:**
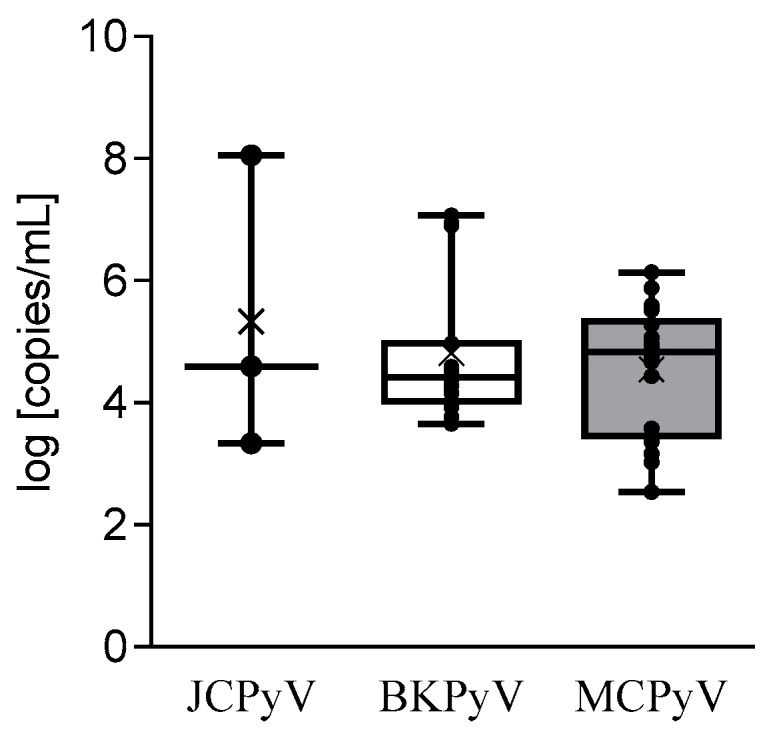
Distribution of the viral load of JCPyV, BKPyV, and MCPyV in the CSF. Each bullet point indicates the viral load of a CSF sample, the x indicates the mean viral load, the middle line indicates the median viral load, and the upper and lower lines indicate the maximum and minimum viral load value for each tested virus.

Figure 1 summarizes the HPyV load results: JCPyV was detected at the highest (*p* < 0.05) mean load (3.7 × 10^7^ copies/mL, range: 2.2 × 10^3^–1.1 × 10^8^ copies/mL), followed by BKPyV (1.9 × 10^6^ copies/mL, range: 4.4 × 10^3^–1.2 × 10^7^), MCPyV (1.9 × 10^5^ copies/mL, range: 3.5 × 10^2^–1.4 × 10^6^ copies/mL), and HPyV-6 (3.3 × 10^4^ copies/mL).

The cycle threshold (CT) for every positive sample is reported in Table S2. For JCPyV, the CT ranged between 23.37 and 39.05, for BKPyV, between 31.45 and 38.76, and for MCPyV, between 33.56 and 39.10.

Additionally, the percentages of infected cells were evaluated: JCPyV infected the highest mean percentage of cells (192.25%, range: 0.05%–576.7%), followed by BKPyV (13.48%, range 0.01%–29.3%), and MCPyV (0.84%, range: 0.01%–3.02%).

### 3.3. HPyV Distribution in the CSF According to the Disease

The patients infected with HPyVs were affected by meningoencephalitis (25), peripheral neuropathy/polyradiculoneuropathy (5), PML (3), Sjogren syndrome (1), cognitive disorder (1), migraine (1), myeloproliferative disease (1), angioma (1), multiple sclerosis (1), mood disorders (1), and brain tumor (1), as seen in Table 4.

### 3.4. HPyV Molecular Characterization

**Figure 2:**
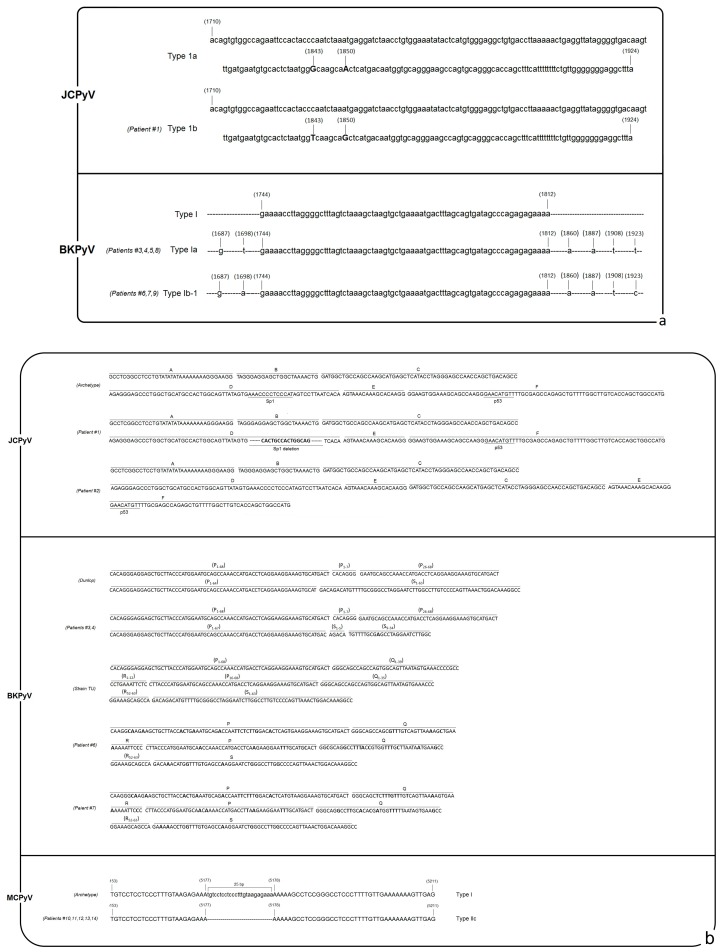
(**a**) Schematic diagram of the JCPyV and BKPyV VP1 sequences amplified in the CSF. The obtained sequences are compared with genotype 1a for JCPyV [38] and with genotype I for BKPyV [41]. Point mutations are highlighted in bold; (**b**) schematic diagram, using the typical block representation, of the JCPyV, BKPyV, and MCPyV NCCR sequences amplified in the CSF. The obtained sequences are compared with the archetype CY (accession number AB038249) for JCPyV, with the Dunlop (accession number V01108) or TU strain (accession number LC29411) for BKPyV, and with Type I for MCPyV (accession number EU375803). Point mutations are highlighted in bold.

Molecular characterization of the HPyV-positive strains was not performed for all the positive CSF samples due to a lack of clinical specimens. Regarding JCPyV, one amplified strain was characterized as 1B and two NCCRs were defined as “rearranged.” BKPyV VP1 genotyping was performed for seven amplicons, Ia (4/7) and Ib-1 (3/7); the BKPyV NCCR was similar but not identical to the BKPyV Dunlop strain in two cases and to the TU strain in two other cases. The MCPyV NCCR was amplified and sequenced in five CSF samples, and it was characterized as an IIc strain, as seen in Figure 2, panels a and b.

## 4. Discussion

HPyVs can be present in different body compartments and/or body fluids. Understanding their tissue tropism may provide insights into the possible pathogenic roles of these viruses in human diseases. In this retrospective study, we investigated the presence of the genome of some HPyVs (JCPyV, BKPyV, MCPyV, HPyV6, HPyV7, and HPyV9) in the CSF of patients with neurological diseases, mostly not related to other microbial agents.

None of the samples analyzed were positive for HPyV7 and HPyV9. HPyV7 has been previously detected in urine and nasopharyngeal swabs of children undergoing liver transplant [42] as well as on the skin [7]. In this district, HPyV7 has been associated with the development of pruritic rashes and dyskeratotic skin disorders in immunocompromised patients [7,21]. HPyV9 has been previously isolated from the serum of a kidney transplant patient and from the skin of a patient with MCC [8,22]. To our knowledge, HPyV9 has not been detected in other biological samples, and our study is in accordance with the original observation of Scuda et al. who did not detect HPyV9 in the CSF of patients with PML [8], suggesting that the CNS is likely not a target of the virus.

As expected, JCPyV genome was detected in the CSF collected from the patients affected with PML. We reported frequent amplifications of BKPyV and MCPyV and a single amplification of the HPyV6 DNA sequences. Even though the prevalence of BKPyV and MCPyV was found to be significantly greater than that of JCPyV, the viral loads and the percentage of cells infected were definitely much higher for JCPyV than the other two HPyVs.

As already stated, this is not the first time that the BKPyV genome has been detected in the CSF or brain tissue. Chittick and colleagues reviewed all the cases of BKPyV isolated from the CNS up to 2013 [43]; subsequently, at least two other cases have been published [44,45]. Additionally, the BKPyV genome was amplified in the CSF from patients with multiple sclerosis and Huntington’s disease, as well as from asymptomatic subjects [46,47]. It is debatable whether BKPyV plays a significant role in the development of CNS disorders; however, it is nonetheless plausible that it might represent a true pathogen, at least when it is detected at high viral loads and/or infected cell percentages. This was the case for two patients from our cohort, with meningoencephalitis and with BKPyV loads in the CSF between 10^6^ and 10^7^ copies/load. In another patient, a high BKPyV load was found in the CSF, but with a concomitant infection of EBV and HSV-1, which were the likely etiological agents of meningoencephalitis. In this case, BKPyV could probably play a copathogenic role or be latent in the CNS, and then occasionally be found in the CSF. Concerning the coinfections, it should be emphasized that two other cases showed concomitant infections of BKPyV and herpesvirus or enterovirus.

Our results are inconsistent with those from previous reports about the presence of MCPyV in the CSF. However, it should be underlined that, so far, only three papers have reported results about the detection of the MCPyV genome in a total of 168 CSF samples [27,30,48]. Moreover, MCPyV has been previously found in a high number of malignant CNS tissues at very low numbers of copies/cell [49]. In contrast, MCPyV is generally present at high copies/cell numbers in MCC tumor cells. Consequently, our findings suggest that MCPyV DNA may be present in its latent state in the CNS or, alternatively, it could reach the CNS from the peripheral district, where it might actively replicate.

Because MCPyV and HPyV6 are part of the human skin microbiota, it is nonetheless possible that the MCPyV and HPyV6 sequences originated from the skin of the patient during CSF collection, as occurs with bacterial contamination of blood after venipuncture [50,51].

Additionally, the presence of the viral genomes in the sera was determined in only one-third of the positive patients and for only MCPyV and BKPyV. In these cases, only BKPyV was found in one serum sample from a patient affected with neuropathy. Besides this single case, in the other patients HPyVs were probably latent in the CNS, and they did not passively cross the blood–brain barrier (BBB). However, since we are considering diseases often characterized by inflammatory alterations of the BBB itself, assessing the presence of HPyVs in the peripheral district of all the enrolled patients would have been of great interest, to understand whether HPyVs cross the BBB or are already present in the CNS.

For a few viral strains, we conducted a molecular characterization. The genotyping results for JCPyV and BKPyV were consistent with those of previously published epidemiological studies, which identified genotypes 1 and I, respectively, as the most represented genotypes within the European population. In addition, the presence of a rearranged NCCR JCPyV infecting PML patients has been widely reported in the literature [52]. The four sequenced BKPyV NCCRs showed different rearrangements and point mutations and were classified arbitrarily as variants of the known Dunlop and TU strains [24]. In contrast to that of JCPyV and BKPyV, the MCPyV NCCR structure has been previously associated with the geographic origin of the patients. We found the MCPyV NCCR IIc strain in the CSF, which has been reported as the predominant strain in specimens from white persons of European descent, as expected for our cohort of patients [37].

## 5. Conclusions

Our study: (a) confirmed the neurotropism of JCPyV; (b) indicated the occasional presence of BKPyV and the high prevalence of MCPyV in the CSF of patients affected with neurological disorders, mostly not associated with other microbial agents; (c) did not find evidence of the presence of HPyV7 and 9, and limited presence of HPyV6, in the CSF.

The strength of the study arises from the fact that, to the best of our knowledge, this is the largest report on HPyVs presence in the CSF. The description of some CNS-infecting viral strains represents an additional benefit of this study, since there are very few data in the literature defining the genotype and/or the rearrangements of HPyVs other than JCPyV in the CSF.

However, the study suffers from some limitations: it would have been more informative to report the results for all the 14 known HPyVs, the data obtained from paired peripheral clinical specimens, such as blood or serum, and the characterization of the sequences of all the amplified viral strains.

Whether it is worth searching for several HPyVs in the CSF from patients affected with neurological diseases is still an unanswered, but significant question. The results of our study might indicate the utility of testing BKPyV and MCPyV in addition to the microbial agents usually tested in meningoencephalitis and other neurological diseases.

## Figures and Tables

**Table 1:** Main demographic and clinical characteristics of the enrolled patients.

Age in years, mean (range)	58 (17–92)
Female, number (%)	117 (50.0%)
Suspected diagnosis at hospital admission	Meningitis/encephalitis, 139 (59.4%) Peripheral neuropathy, 30 (13%) Cognitive disorders, 19 (8.1%) Multiple sclerosis, 10 (4.2%) Brain tumor/metastasis, 9 (3.8%) Epilepsy, 5 (2.1%) Confused state, 5 (2.1%) Hydrocephalus, 4 (1.7%) Migraine, 4 (1.7%) Progressive multifocal leukoencephalopathy, 3 (1.3%) Mood disorder, 3 (1.3%) Myelitis, 3 (1.3%) Sjogren syndrome, 1 (0.4%) Guillain–Barré syndrome, 1 (0.4%) Myeloproliferative disease, 1 (0.4%) Angioma, 1 (0.4%)

**Table 2:** HPyVs DNA prevalence in the cerebrospinal fluid (CSF) of the studied cases.

HPyVs Prevalence					
No. of Cases	JCPyV+/tot (%)	BKPyV+/tot (%)	MCPyV+/tot (%)	HPyV-6+/tot (%)	HPyV+/tot (%)
234	3/234 * (1.3%)	15/234 *° (6.4%)	22/234 *° (9.4%)	1/234 * (0.4)	41/234 (17.5)

* = *p* < 0.05 (MCPyV vs. JCPyV/HPyV6 and BKPyV vs. JCPyV/HPyV6); ° = three patients were BKPyV/MCPyV coinfected.

**Table 3:** Number of cases with coinfections of HPyVs and other viruses.

	JCPyV+	BKPyV+	MCPyV+
**HSV-1+**	/	1	1
**EBV+**	2	2	/
**Enteroviruses+**	/	1	3
**HIV+**	2	/	/

**Table 4:** HPyV distribution in the CSF of patients according to their neurological disease.

HPyVs (no. + Cases)	Disease (Frequency; Percentage)
JCPyV + (3)	PML (3/3; 100%)
BKPyV + (15)	Meningoencephalitis (9/135; 6.7%) Peripheral neuropathy (3/30; 10%) Cognitive disorder (1/19; 5.3%) Sjogren syndrome (1/1; 100%) Mood disorder (1/3; 33%)
MCPyV+ (22)	Meningoencephalitis (15/135; 11.1%) Neuropathy (2/30; 6.7%) Migraine (1/4; 25%) Multiple sclerosis (1/10; 10%) Brain tumor (1/9; 11.1%) Myeloproliferative disease (1/1; 100%) Angioma (1/1; 100%)
HPyV6+ (1)	Meningoencephalitis (1/135; 7.4%)

## Supplementary Materials

Supplementary materials can be found at https://www.mdpi.com/2076-2607/8/1/16/s1.
