# Supplementary material for: Human Polyomaviruses in the Cerebrospinal Fluid of Neurological Patients
Source: Microorganisms. 2019 Dec 20;8(1):16. doi: 10.3390/microorganisms8010016 (PMC7022863; doi:10.3390/microorganisms8010016)
Supplement: Supplementary file 1 [file microorganisms-08-00016-s001.pdf]

Table S1: Primer and probe sequences employed for the search of the HPyV genomes in the CSF.

VIRUS	TARGET REGION	nt	PRIMERS/ PROBE	SEQUENCE
**JCPyV**	Large T Antigen	4299–4321*	JCV Forward	5′-GAGTGTTGGGATCCTGTGTTTTC-3′
		4352–4375*	JCV Reverse	5′-GAGAAGTGGGATGAAGACCTGTTT -3′
		4323–4350*	JCV Probe	5′-FAM-TCA TCA CTG GCA AAC ATT TCT TCA TGG C-MGB-3′
**BKPyV**	VP1	2511–2531°	BKV Forward	5′-AGT GGA TGG GCA GCC TAT GTA-3′
		2586–2605°	BKV Reverse	5′-TCA TAT CTG GGT CCC CTG GA -3′
		2535–2556°	BKV-Probe	5′VIC-TAT GGA ATC CCA GGT AGA AGA-MGB 3′
**MCPyV**	VP1	4053–4072**	MCV Forward	5′-TGCCTCCCACATCTGCAAT -3′
		4090–4112**	MCV Reverse	5′-GTGTCTCTGCCAATGCTAAATGA -3′
		4074–4089**	MCV Probe	5′-FAM-TGT CAC AGG TAA TAT C -MGB-3′
**HPyV6**	VP1	1767–1786 +	6 F	5′-GGCCTGGAAGGGCCTAGTAA -3′
		1847–1823 +	6 R	5′-ATTGGCAGCTGTAACTTGTTTTCTG -3′
		1789–1806 +	6 Probe	5′-JOE-AGA ACC AAC CAT CTG TTG- BHQ1 -3′
**HPyV7**	VP1	1774–1796^	7 F	5′-AGGTCAATGAAGCCCTAGAAGGT-3′
		1840–1822^	7 R	5′-TGCTTTCTGAGGGCTTGCA-3′
		1798–1817^	7 Probe	5′-FAM-CAG GCA ATA CTG ATG TAG C-MGB-3′

**HPyV9**	VP1	1449–1469[#]	9 F	5′-CCCCAAAGAAAAGGCAAGAG -3′
		1509–1493[#]	9 R	5′-GCGGGTGTTGGACAGGTTT-3′
		1472–1488[#]	9 Probe	5′-VIC-CGG AGC ATG TCC TGT AA-MGB-3′

Legend: [*]Reference strain: MAD 1 (accession number J02226.1), [°]Reference strain: WW (accession number AB211371), [**]Reference strain: MCC 350 (accession number EU375803), [+] Reference strain: isolate 627a, complete genome (accession number HM011563), [^] Reference strain: isolate 713a; complete genome (accession number HM011566), [#] Reference strain: isolate HPyV9 complete genome (accession number HQ696595).

Table 2. CT values of the CSF samples positive for HPyV.

	CSF sample	CT	Viral load (copies/mL)
**JCPyV**	LR 8	23.37	1.12×10^8
	LR 23	34.85	3.97×10^4
	LR 117	39.05	2.16×10^3
**BKPyV**	LP25	38.16	1.90×10^4
	LP31	38.76	1.46×10^4
	LP36	37	3.17×10^4
	LP37	37.61	2.43×10^4
	LP40	34.55	9.41×10^4
	LP41	36.54	3.89×10^4
	LP56	36.79	3.49×10^4
	LR 32	38.4	4.43×10^3
	LR 36	36.94	1.07×10^4
	LR 41	35.44	2.64×10^4
	LR 48	37.33	8.47×10^3
	LR 52	37.96	5.77×10^3
	LR 106	31.79	8.87×10^6
	LR 107	31.45	8.0×10^6
	LR 111	32.74	1.18×10^7
**MCPyV**	LP4	36.22	3.32×10^5
	LP17	37.28	1.46×10^3
	LP22	38.7	3.46×10^2
	LP23	37.94	1.06×10^3
	LP36	36.72	1.14×10^5
	LR 21	37.15	3.79×10^3
	LR 22	34.85	2.78×10^4
	LR 36	37.8	2.83×10^3
	LR 40	37.46	2.88×10^3
	LR 41	37.71	2.31×10^3
	LR 70	34.25	4.68×10^4
	LR 105	33.56	1.37×10^6
	LR119	39.25	5.33×10^4
	LR123	38.49	8.67×10^4
	LR127	36.94	1.92×10^5
	LR137	35.12	7.63×10^5
	LR138	34.96	4.0×10^5
	LR150	38.36	9.43×10^4
	LR158	39.1	5.87×10^4
	LR159	35.03	3.83×10^5
	LR162	38.05	8.20×10^4
	LR176	38.12	8.39×10^4
**HPyV6**	LR51	38.7	3.3×10^4

Other Viruses Detected by PCR

HSV-1 was detected in one sample with a viral load of 3.8×10^3 copies/mL (HSV-1 ELITe MGB kit, Elitech, Tourin, Italy), corresponding to a CT value of 31. It was also detected in two samples with a viral load below the limit of quantification of our assay ($<1.1 \times 10^2$ copies/mL), corresponding to a CT value of 39.

EBV positive samples (8) had a viral load below the limit of quantification of our assay ($<1.1 \times 10^2$ copies/mL), EBV ELITe MGB kit, Elitech, Tourin, Italy), corresponding to a CT value of 39.

HCMV was detected in one sample, with a viral load of 7.6×10^3 copies/mL (CMV ELITe MGB kit, Tourin, Italy), corresponding to a CT value of 28.

Enterovirus genomes (3) were detected by a qualitative assay (Enterovirus R-Gene®, Biomerieux, Italy). The CT values ranged from 38 to 40.

HIV-1 was detected with the COBAS® AmpliPrep/COBAS® (TaqMan® HIV-1 Test, v2.0 Roche Diagnostics). The following viral loads were measured: <20 copies/mL, 9×10^2 copies/mL, 3.8×10^3 copies/mL, and 1.5×10^4 copies/mL. We cannot provide the CT value, because the system does not allow the operator to access this kind of information.

The CT of the negative control referred to the internal control. The CT value for the internal control was 25–26.
